# Trends in glaucoma practices among a cohort of ophthalmologists in Latin America: A survey-based study

**DOI:** 10.1038/s41433-025-04123-3

**Published:** 2025-12-09

**Authors:** Alejandro Marin, Sebastian Lacau, Junior P. Linarez, Elena Bitrian

**Affiliations:** Department of Ophthalmology, Bascom Palmer Eye Institute, Miami, Florida USA

**Keywords:** Business and industry, Technology

## Abstract

**Purpose:**

To descriptively assess clinical preferences, practice patterns, and demographic characteristics related to glaucoma care among a cohort of ophthalmologists in Latin America, through a structured survey focusing on intraocular pressure measurement methods, visual field testing, and work-related perceptions.

**Methods:**

A cross-sectional survey was conducted to assess the clinical practices of ophthalmologists who attended the XLVI Inter-American Course in Clinical Ophthalmology (CURSO) at the Bascom Palmer Eye Institute. The survey collected demographic and professional data, as well as information on practice patterns, preferences, and beliefs related to glaucoma evaluation. The responses were categorised and analysed to identify trends in clinical practices.

**Results:**

In a survey of 142 Latin American ophthalmologists attending the XLVI Inter-American CURSO, the Goldmann tonometer was the most used, preferred, and trusted method for IOP measurement. Humphrey visual fields were the predominant tool for perimetry. Sixty-three percent reported having an assistant to assess basic parameters like visual acuity and IOP. Most participants (90%) worked in the private sector; 51% considered their compensation fair, while 27% felt undercompensated. Retirement age expectations varied, with most planning to work until at least age 70.

**Conclusion:**

Glaucoma evaluation results from this survey may reflect Latin American practice patterns. Based on this assumption, Goldmann and Humphrey are the most popular tools for measuring IOP and conducting perimetry, ophthalmic assistants often assess basic eye parameters, and ophthalmologists report varying satisfaction with their income and retirement age.

## Introduction

According to Stamper (2011), for over a millennium, physicians have suspected that intraocular pressure (IOP) is related to some form of blindness. Various reports claimed that an increase in IOP could lead to an irreversible form of blindness. However, it was not until the late 19th century that elevated IOP was widely accepted as a distinctive characteristic of glaucoma.

The first instrument for measuring IOP indirectly was developed in 1863 by Von Graefe. This device consisted of a tonometer that measured intraocular pressure using a weighted plunger, which determined the indentation of the sclera. Although multiple indentation tonometers were later invented, in 1905 Hjalmar Schiotz created a transcorneal indentation tonometer that provided significantly more accurate IOP measurements.

The Schiotz tonometer had several positive characteristics: it was made of metal, allowing for sterilisation in an autoclave, and it was portable. However, limitations included the necessity of supine position and risk for corneal abrasion if patients moved their eyes during measurements. Despite its disadvantages, the Schiotz tonometer was widely used throughout much of the 20th century until it was replaced in the last quarter by the Goldmann tonometer [1].

The Goldmann tonometer is based on the Imbert-Fick principle, which states that in an ideal, dry, thin-walled sphere, the internal pressure of the sphere is equal to applied force divided by the flattened area. This principle allows the deduction of intraocular pressure, as it is proportional to the force applied over an area of the eyeball (the cornea) and the thickness of its wall (the thickness of the flattened cornea). Since its implementation, the Goldmann tonometer has become the most widely used instrument for measuring IOP due to its affordability, reproducibility, and accuracy. Variants of the Goldmann tonometer, such as the Perkins and Draeger tonometers, allow for measurements in a supine position, making them particularly useful for bedridden patients or those under general anaesthesia [1, 2].

Since Goldmann tonometers require contact, non-contact or reduced-contact tonometers emerged in the second half of the 20th century as alternatives that have the advantage of reducing risk for infections and corneal abrasions, offering comparable accuracy to that of the Goldmann tonometer [3]. Today, a wide variety of tonometers are available, but to date, no studies have analysed trends in tonometer usage and glaucoma evaluation among ophthalmologists in Latin America.

Perimetry, the systematic measurement of visual fields, an important tool to assess glaucoma, has also undergone significant evolution since its inception. Von Graefe, in his work ‘Examination of the Visual Functions in Amblyopic Affections’, introduced perimetry into clinical medicine. He identified various defects, including ring scotomata, concentric constriction, and hemianopias, and suggested their neurological causes. Following von Graefe, Jannik Bjerrum popularised campimetry by constructing a 2-m tangent screen and using different target sizes to generate multiple isoptres, introducing coloured stimuli to enhance testing [4].

The late 20th century marked a significant leap with the advent of automated perimetry. The Octopus perimeter was developed in the late 1960s to early 1970s and was the first automated device for visual field testing. Anders Heijl contributed to the development of the Humphrey Field Analyzer, which further refined automated testing [4]. The Humphrey Visual Perimeter is considered the gold standard, and most glaucoma clinical trials have relied upon it for their visual field measurement [5–11]. Recently, virtual reality perimetry testing has also received increased attention in the last few years because of its ability to create an immersive site for testing, which minimises distractions while being portable and affordable [12], but its widespread usage among Latin American ophthalmologists remains poorly known.

Ophthalmologist job satisfaction worldwide is a dynamic parameter that differs across regions. A study performed in the University of Miami, using the AAO database, concluded that among ophthalmologists who recently concluded training, 83% reported being happy with work life [13], while another survey conducted by the AAO in 2017 reports a satisfaction rate of 93% [14]. Another survey among national panellists in 2003 stated that 52% of the panellists wanted to retire between ages 65, and 70, and 35% before 65 [15], and a more recent one among chairs of academic departments in ophthalmology in the United States of America indicates that 86% would like to retire after age 70 [15].

The objective of this publication is to evaluate the demographic profile, clinical practices, and professional perceptions of ophthalmologists attending the XLVI Inter-American CURSO, with a particular focus on glaucoma-related diagnostic methods such as intraocular pressure measurement and visual field testing. Since our primary target was Latin American and Caribbean ophthalmologists, CURSO was selected as the ideal conference to conduct this survey. The event is specifically designed for practicing ophthalmologists from these regions, who gather in Miami for this annual meeting, organised by the Bascom Palmer Eye Institute, and offered in both English and Spanish [16]. While previous large-scale surveys have explored subspecialty practice this region of interest, such as the ocular surface survey by Borrone et al. [17], to our knowledge, this is the first survey focused specifically on glaucoma practice patterns in this population.

## Methodology

A cross-sectional survey was designed using Google Forms to assess participant perspectives. The survey was promoted through a strategically placed QR code within the course venue and its surroundings. Participation was voluntary and anonymous. Before responding, participants were presented with an informed consent statement outlining the purpose of the study, the voluntary nature of participation, and assurances of confidentiality. By proceeding with the survey, participants indicated their consent to participate. To encourage participation, respondents were offered the opportunity to enter a raffle for a gift valued at $150 by submitting their email address through a separate, unlinked form to ensure anonymity. This study did not require IRB approval as no identifiable personal or clinical data were collected, and participation was entirely anonymous and voluntary.

By scanning the QR code, participants accessed the survey and had the option to choose between English or Spanish to continue with the survey. The questionnaire included the following questions:

1. Name (open-ended)
2. Surname (open-ended)
3. Gender (single-choice: male, female, other)
4. Age (open-ended)
5. Country of ophthalmology practice (open-ended)
6. Field of activity (multiple-choice: private clinic practice, public hospital practice, basic science research (laboratory), ophthalmology residency applicant, none of the above).
7. Ophthalmology subspecialty (multiple-choice: comprehensive ophthalmology, anterior segment, glaucoma, medical retina, surgical retina, oculoplastics, paediatric ophthalmology & strabismus, neuro-ophthalmology, uveitis, oncology & genetics).
8. Years of experience in ophthalmology practice (single-choice: 0-5, 6-10, 11-15, 16-25, 25 + , resident, not applicable)
9. Most commonly used method for measuring intraocular pressure (single-choice: Goldmann tonometry, Perkins tonometry, pneumotonometry, dynamic contour tonometry (PASCAL), electronic indentation/applanation tonometry (TonoPen), rebound tonometry (iCare), indentation tonometry (Schiötz))
10. Preferred method for measuring intraocular pressure (same options as previous question)
11. Second preferred method for measuring intraocular pressure (same options as previous question)
12. Most reliable method for measuring intraocular pressure according to your experience (same options as previous question)
13. Do you have personnel assisting with visual acuity and intraocular pressure measurement? (single-choice: yes, no)
14. If yes, which method does your staff use to measure intraocular pressure? (single-choice: same options as question 9, plus “Not applicable”)
15. Primary method used for visual field testing (single-choice: Humphrey, Octopus, virtual reality set)
16. Perception of income in relation to work (single choice: my remuneration is excellent, my remuneration is fair, I am underpaid)
17. Until what age do you plan to practice as an ophthalmologist? (open-ended)

Personal information collected (name and surname) was used exclusively for identifying participants for prise distribution. Question 6 allowed for the exclusion of non-ophthalmologists or non-resident participants, although exclusion in this study did not invalidate their participation in the raffle.

The collected data was exported and analysed by the authors to identify trends in glaucoma-related practices among the course attendees.

## Results

Out of the six hundred participants at the CURSO event, a total of 142 practicing ophthalmologists from various regions of Latin America responded the survey. Notable participation was observed from Colombia (25 respondents, 18%), Bolivia (21 respondents, 15%), and Peru (19 respondents, 13%) (Table 1). The majority of respondents were male (55.63%), while female participants accounted for 63 responses (44.37%).

**Table 1:** Geographic distribution of responders by country.

Country	Number of participants	%
Argentina	6	4%
Barbados	1	1%
Bolivia	21	15%
Brazil	1	1%
Chile	2	1%
Colombia	25	18%
Costa Rica	6	4%
Ecuador	15	11%
El Salvador	5	4%
Spain	1	1%
Guatemala	8	6%
Haiti	5	4%
Honduras	1	1%
Mexico	6	4%
Nicaragua	3	2%
Panama	1	1%
Paraguay	2	1%
Peru	19	13%
Dominican Republic	8	6%
Trinidad	1	1%
United States	1	1%
Venezuela	4	3%

The majority of respondents (93.0%) completed the survey in Spanish. Among the 10 who chose English, 2 were from Spanish-speaking countries (Bolivia and the Dominican Republic), 5 from a French-speaking country (Haiti), 1 from a Portuguese-speaking country (Brazil), and 2 from English-speaking countries (Trinidad and Barbados).

**Fig. 1:**
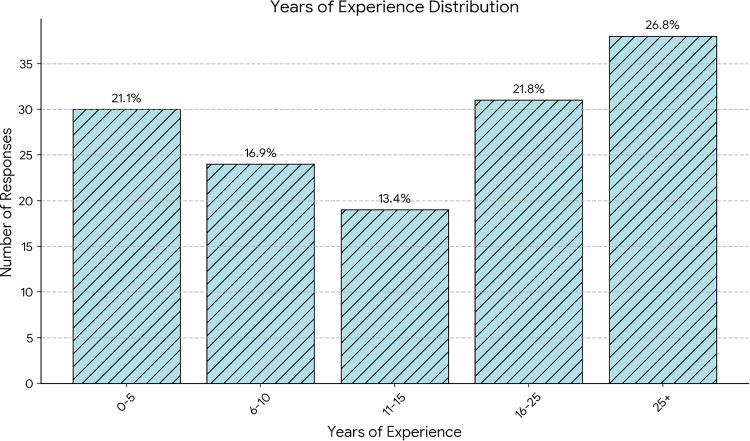
Distribution of responders by years of experience.

The survey revealed that 57% of respondents reported work exclusively in private clinics, 10% worked solely in public hospitals, and 33% reported working in both private clinics and public hospitals. As shown in Fig. 1, participants had a diverse range of experience in ophthalmology: 21% had less than five years of experience, while 26% had over 25 years of professional practice.

**Fig. 2:**
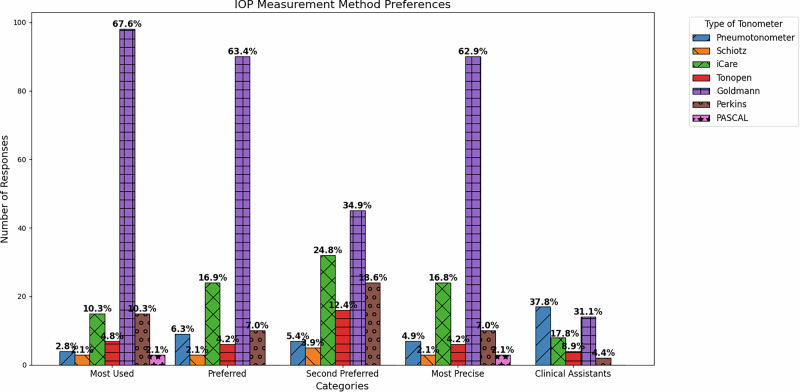
Tonometry responses: Most Used, Preferred, Second Preferred, Considered Most Precise, and Clinical Assistant Usage.

Goldmann tonometry was the most used method for measuring IOP (69%), followed by rebound tonometry and Perkins tonometry (both 10%). Additionally, Goldmann tonometry was the preferred method for 63% of respondents, followed by rebound tonometry (iCare) at 17%. However, when asked about their second preferred method, devices such as the Perkins tonometer, rebound tonometry, pneumotonometry, and Tono-Pen gained more relevance. Regarding the most used method for IOP measurement, the Goldmann tonometer led with a 69% preference, followed by rebound tonometry and the Perkins tonometer, both at 10%. Additionally, Goldmann tonometry was selected as the preferred method by 63% of respondents, followed by rebound tonometry (iCare), which received 17% of the votes. However, when asked about their second favourite method for measuring IOP, devices such as the Perkins tonometer, rebound tonometer, non-contact tonometer, and Tono-Pen gained greater relevance. Finally, an overwhelming 93% of respondents considered the Goldmann tonometer to be the most reliable method for measuring IOP, followed by the Perkins tonometer, with 6% of the votes (Fig. 2).

Regarding clinical assistance, 63% of respondents indicated that they did not have help with measuring parameters such as visual acuity or IOP, while 37% stated that they did. Among the 52 physicians who responded affirmatively, 45 (86%) indicated that their assistants primarily used the non-contact tonometer to measure IOP (38%), followed by the Goldmann tonometer (31%) (Fig. 2).

Regarding visual field evaluation, the predominant method was Humphrey perimetry, used by 83% of respondents. The Octopus perimeter was selected by 14% of respondents, and virtual reality devices by 2%.

Finally, on a more personal level, out of the 142 participants, 72 (51%) considered their compensation to be fair, 19 (13%) rated it as excellent, 12 (8%) preferred not to respond, and 39 (27%) indicated that they were not well compensated. Regarding retirement plans, 15 (10%) participants expressed their desire to work beyond the age of 80, 58 (40%) until ages 70–79, 50 until ages 60–69, 5 (3%) until ages 50–59, and 11 (7%) mentioned that their decision depended on factors such as health, retirement benefits, and compensation.

## Discussion

This survey highlights that, despite the Goldmann tonometer being over half a century old since its introduction, it remains the preferred, most used, and is perceived as the most precise method for measuring IOP among ophthalmologists attending the XLVI Bascom Palmer Course in Latin America. The dominance of Goldmann tonometry likely originates from its well-established validation in clinical research, widespread adoption in practice guidelines, and its role as the historical gold standard for IOP measurement. Studies consistently emphasise its reproducibility, accuracy, and reliability as the gold standard in tonometry [1].

The survey also revealed a discrepancy between ophthalmologists’ preferences and the methods employed by clinical assistants. While ophthalmologists favoured Goldmann tonometry, assistants used more frequently non-contact tonometers or rebound tonometry. This divergence may reflect practical considerations in clinical workflow: non-contact devices and rebound tonometry require less technical expertise.

Next, the strong preference for Humphrey perimetry (83%) aligns with global trends, as this method remains as it remains widely regarded as the clinical standard against which other devices are evaluated [18]. The limited adoption of virtual reality-based perimetry (2%) may suggest either scepticism toward emerging technologies or challenges such as institutional inertia and limited clinical validation within the Latin American context.

In addition, 93% of respondents completed the survey in Spanish, only one participant from a non-speaking country that has no Spanish as is official language (United States) responded in Spanish rather than English. This suggests that Latin American ophthalmologist feel more comfortable and prefer to engage in Spanish-language activities, indicating a strong preference for Spanish communication within this cohort when given the option.

Beyond clinical practices, 64% of participants reported their remuneration as fair or excellent, and the majority of responders planned to practice until at least 70 years old. These results may be explained by factors such as job satisfaction, financial incentives, and personal motivations.

### Limitations

This study has several limitations. Although the sample included participants from various regions of Latin America, it was not fully representative of the region. Most responses came from Colombia (18%), Bolivia (15%), and Peru (13%), while participation from other countries was minimal (ranging from 1% to 6%), potentially limiting the generalisability of the findings across Latin America. Additionally, the sample, though diverse, was drawn from attendees of a single academic course, potentially introducing selection bias toward practitioners with specialised interests or access to continuing education. Moreover, the use of self-reported data may be subject to recall bias or reporting inaccuracies regarding clinical practices. Finally, subgroup analyses were not performed due to the small sample sizes within each subgroup.

## Conclusion

The results of the glaucoma evaluation from this survey, conducted during the XLVI Bascom Palmer Inter-American course, may reflect glaucoma practice patterns in Latin America, although they have limitations. Goldmann applanation tonometry and Humphrey perimetry appear to be the most popular tools respectively for measuring IOP and performing visual field testing. The use of assistants to measure basic parameters, such as visual acuity and IOP, may be common in Latin America. Additionally, ophthalmologists in the region could have varying levels of satisfaction with their income and their age of retirement.

## Summary

### What was known before

- Goldmann tonometry and automated perimetry were the gold standards for glaucoma evaluation, but regional usage patterns in Latin America were not well studied.

### What this study adds

- It identifies current trends and preferences in glaucoma assessment tools among a Latin American cohort of ophthalmologists and trainees, confirming Goldmann tonometry and Humphrey perimetry as the most commonly used methods.

## Data Availability

The datasets generated and analysed during the current study are available from the corresponding author on reasonable request.
